# Investigating the modulation of genetic effects on late AMD by age and sex: Lessons learned and two additional loci

**DOI:** 10.1371/journal.pone.0194321

**Published:** 2018-03-12

**Authors:** Thomas W. Winkler, Caroline Brandl, Felix Grassmann, Mathias Gorski, Klaus Stark, Julika Loss, Bernhard H. F. Weber, Iris M. Heid, for the International Age-related Macular Degeneration Genomics Consortium (IAMDGC)

**Affiliations:** 1 Department of Genetic Epidemiology, University of Regensburg, Regensburg, Germany; 2 Department of Ophthalmology, University Hospital Regensburg, Regensburg, Germany; 3 Institute of Human Genetics, University of Regensburg, Regensburg, Germany; 4 Medical Sociology, Institute of Epidemiology and Preventive Medicine, University of Regensburg, Regensburg, Germany; University of Manchester, UNITED KINGDOM

## Abstract

Late-stage age-related macular degeneration (AMD) is the leading cause of visual impairment in the elderly with a complex etiology. The most important non-modifiable risk factors for onset and progression of late AMD are age and genetic risk factors, however, little is known about the interplay between genetics and age or sex. Here, we conducted a large-scale age- and sex-stratified genome-wide association study (GWAS) using 1000 Genomes imputed genome-wide and ExomeChip data (>12 million variants). The data were established by the International Age-related Macular Degeneration Genomics Consortium (IAMDGC) from 16,144 late AMD cases and 17,832 controls. Our systematic search for interaction effects yielded significantly stronger effects among younger individuals at two known AMD loci (near *CFH* and *ARMS2/HTRA1*). Accounting for age and gene-age interaction using a joint test identified two additional AMD loci compared to the previous main effect scan. One of these two is a novel AMD GWAS locus, near the retinal clusterin-like protein (*CLUL1*) gene, and the other, near the retinaldehyde binding protein 1 (*RLBP1*), was recently identified in a joint analysis of nuclear and mitochondrial variants. Despite considerable power in our data, neither sex-dependent effects nor effects with opposite directions between younger and older individuals were observed. This is the first genome-wide interaction study to incorporate age, sex and their interaction with genetic effects for late AMD. Results diminish the potential for a role of sex in the etiology of late AMD yet highlight the importance and existence of age-dependent genetic effects.

## Introduction

Age-related macular degeneration (AMD) is a degenerative disorder of the central retina and late stage AMD represents the leading cause of irreversible vision loss in the elderly population of western societies [1–3]. Late AMD can present as a neovascular complication, characterized by choroidal/sub-retinal neovascularization (NV), or an atrophicform, known as geographic atrophy (GA) of the retinal pigment epithelium (RPE) [2,3]. Both conditions lead to photoreceptor loss, however, the pathogenesis is only imprecisely understood and therapeutic options are still limited [2–4].

Multiple factors have been shown to play a role in the pathophysiology of this complex disease. Advanced age reveals the strongest association with AMD onset and progression in all population-based or case-control studies [2,3,5,6]. Late AMD develops primarily in individuals aged 70 years and older [2,3]. Sex as another potential risk factor has been debated for many years [3]: While some studies have implicated female sex as an independent risk factor [5,7,8], some have not [9–11], and some have shown the opposite [12]. Furthermore, there exists a strong genetic influence on AMD, which was demonstrated to account for an estimated 50% of late AMD cases [2,13,14]. Some work demonstrated interaction between genetic and non-genetic factors like smoking, chronic infection [15,16], or body mass index [17] on AMD risk. However, adequately powered systematic genome-wide searches for gene-environment interaction (GxE) for AMD are lacking.

The International AMD Genomics Consortium (IAMDGC) has established the largest dataset on the genetics of late AMD with 16,144 late AMD patients and 17,832 controls of European ancestry available to date. In these data, 52 independently associated common and rare genetic variants distributed across 34 genetic loci were identified [13]. With regard to biological insight, the genes underlying these loci were found to be enriched for those involved in the alternative complement pathway, HDL transport, and the extracellular matrix organization and assembly [13]. So far, there is no study investigating whether and to what extent the genetic effects modulating AMD risk are influenced by age or sex. Evaluating sex differences in the genetic effects of AMD could shed light on the role of sex as a risk factor by clarifying whether the ~47% of disease etiology explained by genetics [13] bare sex-differences. An accounting of age and sex and their potential interaction with genetic effects (GxAGE or GxSEX) may increase the statistical power in the search of main genetic effects [18]. Therefore, we set out to investigate the role of age and sex as modulators in the genetics of late AMD in the IAMDGC data and to explore whether new genetic loci for late AMD can be detected when accounting for potential modulators such as age and sex.

## Results

### Effects sizes at the CFH and ARMS/HTRA1 AMD risk loci are more pronounced in the younger

**Fig 1:**
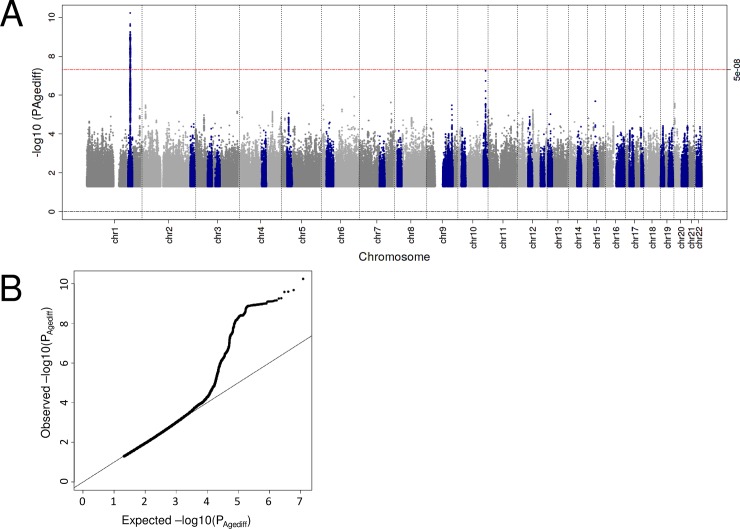
Manhattan and QQ plot of age-difference P-values. Shown are the age-difference P-Values for late AMD by their position on the genome (A, Manhattan plot) as well as their distribution (B, QQ plot). The 34 known genetic regions identified by Fritsche et al [13] are colored blue in the Manhattan plot.

To understand whether genetic effects for late AMD are modulated by age, we conducted age-stratified Firth-corrected logistic regression analyses on AMD for each of the 1000 Genomes-imputed variants in the IAMDGC data set (16,144 patients and 17,832 controls of European ancestry, **Online Methods**). We stratified the full data set by median age among cases and controls separately yielding 7,959 younger cases (≤ 77.8y), 9,072 younger controls (≤ 71.0y), 7,934 older cases (> 77.8y) and 8,653 older controls (> 71.0y). We tested each variant for age differences of the genetic effects (**Online Methods**). This genome-wide scan for age difference (judged at genome-wide significance, P_Agediff_ < 5 x 10^−8^) revealed a single signal with significantly stronger effects among younger compared to older individuals at the *CFH* locus (lead variant rs10922095, OR_younger_ = 2.28, OR_older_ = 1.81, P_Agediff_ = 5.91 x 10^−11^, **Fig 1, Table 1**). This strategy revealed no novel AMD-associated locus. By testing the previously established 34 AMD lead variants for age difference (at Bonferroni-corrected significance, P_Agediff_ < 0.05/34), we identified stronger effects among younger individuals for two variants, including the *CFH* and *ARMS2/HTRA1* loci (rs10922109 and rs3750846, P_Agediff_ = 1.36 x 10^−3^ and 1.04 x 10^−3^, respectively, **Table 1**, **S1 Table**). None of the 34 lead variants exhibited an effect only in one age-group (P_younger_ or P_older_ ≥ 0.05) or effects in opposite directions. A sensitivity analysis comparing genetic effects between truly young (≤65.0y, N = 1,543) and truly old cases (≥85.0y, N = 2,668) yielded a consistent pattern of age-dependent genetic effects on AMD for the highlighted *CFH* and *ARMS2/HTRA1* variants (**S2 Table**). Altogether, we find modulating effects of age on late AMD genetics, identifying three variants in the *CFH* and *ARMS/HTRA1* loci with stronger effects in younger individuals, but no evidence for effects that are protective in one age-group and adverse or zero in the other.

**Table 1:** Two known loci with significant age-difference in genetic effects on late stage AMD. Shown are the genome-wide significant (P_Agediff_ < 5 x 10^−8^) lead variant at the CFH locus and two of the 34 known variants from Fritsche et al [13], which revealed significant age-dependency (P_Agediff_ < 0.05/34, corrected for 34 known lead variants from Fritsche et al). Age-stratified analyses included 17,031 younger (7,959 cases, 9,072 controls) and 16,587 older (7,934 cases, 8,653 controls) individuals.

					Younger Individuals (Cases ≤ 77.8y, Ctrls ≤ 71.0y)				Older Individuals (Cases > 77.8y, Ctrls > 71.0y)				
					EAF				EAF				
rsid	chr:pos	Locus	Known	EA/OA	Cases	Ctrls	OR	CI	Cases	Ctrls	OR	CI	PAgediff
rs10922095	1:196662031	*CFH*		C/T	0.70	0.52	2.29	[2.17;2.41]	0.65	0.52	1.81	[1.72;1.9]	5.91E-11
rs10922109	1:196704632	*CFH*	yes	C/A	0.79	0.58	2.81	[2.67;2.97]	0.76	0.57	2.50	[2.37;2.63]	1.36E-03
rs3750846	10:124215565	*ARMS2/HTRA1*	yes	C/T	0.47	0.21	2.97	[2.83;3.13]	0.40	0.20	2.64	[2.51;2.78]	1.04E-03

Abbreviations: y = years; EA = effect allele; EAF = effect allele frequency; rsid = dbSNP identifier; Chr = chromosome; Pos = position (build 37); OR = odds ratio; CI = confidence interval; PAgediff = P-values for age-difference

### Accounting for age differences reveals two additional AMD loci

**Fig 2:**
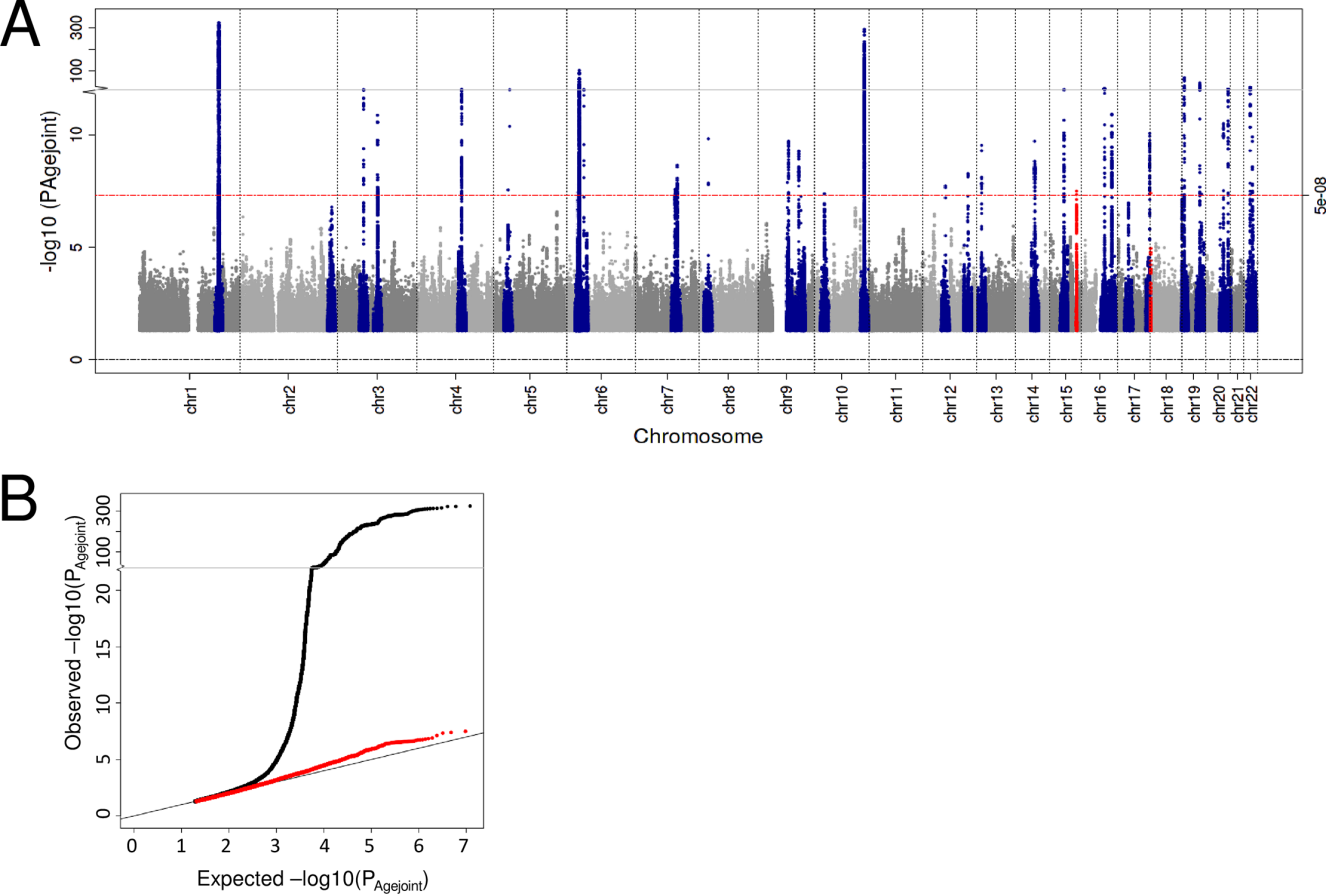
Manhattan and QQ plot of joint age-stratified 2df P-values. Shown are the age-joint P-Values (P_Agejoint_) for late AMD by their position on the genome (Manhattan plot) as well as their distribution (QQ plot). In the Manhattan plot, the 34 known genetic regions identified by Fritsche et al [13] are colored blue and additional genome-wide significant signals are colored red. The QQ plot shows the distribution of P_Agejoint_ including all variants (black) as well as after exclusion of known loci (34 variants +/-500kb, red).

**Fig 3:**
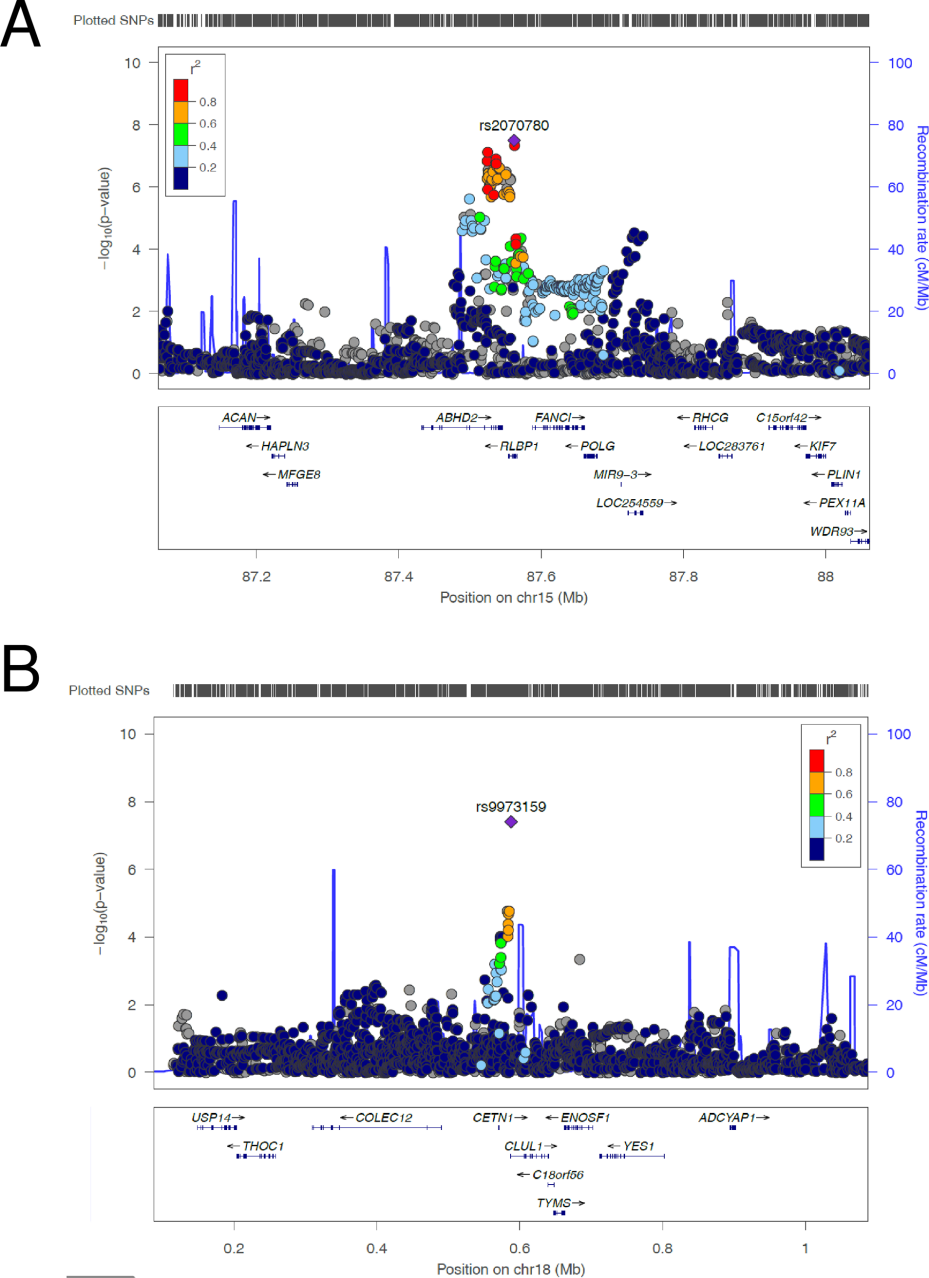
LD region plots of two AMD loci with genome-wide significant joint 2df age-stratified P-Values that were undetected by Fritsche et al. Shown are the age-joint P-Values (P_Agejoint_) for late AMD by their position on chromosome 15 and chromosome 18. The figures were created using Locuszoom (**http://locuszoom.sph.umich.edu/**).

Generally, a search for genetic association variants in late AMD has not considered a potentially modulating effect of age on the genetic effect [13]. A screen which would account for this, e.g. by using the 2 degrees of freedom (2df) joint test and age-stratified effect estimates, can increase the statistical power to detect late AMD genetics [19]. Our genome-wide screen for 2df joint age-stratified effects (judged at genome-wide significance, P_Agejoint_ < 5 x 10^−8^, **Online Methods**) identified 29 independent, significant variants. While 27 of the 29 loci overlap with regions that were identified in the previous screen for AMD (using the identical data set) [13], two additional AMD loci were identified in this study by accounting for age differences. One hit is located in a novel AMD region on chromosome 18 (rs9973159, P_Agejoint_ = 3.91 x 10^−8^) and one in a region on chromosome 15 that was recently identified for AMD in a joint analysis of nuclear and mitochondrial variants [20] (rs2070780, P_Agejoint_ = 3.19 x 10^−8^, **Figs 2 and 3**, **Table 2, S3 Table**). A search for independent second signals at the two loci by conditioning on the two lead variants did not reveal any independent second signals (P_Agejoin;cond_ ≥ 5 x 10^−8^). For each of the two variants, effects were stronger in younger compared to older individuals (rs2070780: OR_Younger_ = 1.13, OR_Older_ = 1.05, P_Agediff_ = 0.019; rs9973159: OR_Younger_ = 1.19, OR_Older_ = 1.09, P_Agediff_ = 0.052). The identification of novel loci with small gene-age interaction effects illustrates the ability of the joint test to leverage potential interactions. The two novel AMD loci were missed in previous studies as these failed to account for gene-age interactions [13].

**Table 2:** Two loci with genome-wide significant age-joint effects on late AMD which were undetected by Fritsche et al. Shown are the two lead variants with genome-wide significant joint-effects on late AMD (P_Agejoint_ < 5 x 10^−8^) for the two loci that were not detected in the previous genome-wide screen by Fritsche et al [13]. Age-stratified analyses included 17,031 younger (7,959 cases, 9,072 controls) and 16,587 older (7,934 cases, 8,653 controls) individuals.

				Younger Individuals (Cases ≤ 77.8y, Ctrls ≤ 71.0y)					Older Individuals (Cases > 77.8y, Ctrls > 71.0y)						
				EAF					EAF						
rsid	Chr	Pos (b37)	EA/OA	Cases	Controls	OR	CI	P	Cases	Controls	OR	CI	P	PAgediff	PAgejoint
rs2070780	15	89760997	T/C	0.50	0.47	1.13	[1.08;1.18]	5.29E-08	0.49	0.48	1.05	[1.01;1.10]	0.0258	0.019	3.19E-08
rs9973159	18	597950	C/T	0.88	0.86	1.19	[1.12;1.28]	1.78E-07	0.87	0.86	1.09	[1.12;1.16]	0.0079	0.052	3.91E-08

Abbreviations: y = years; EA = effect allele; EAF = effect allele frequency; rsid = dbSNP identifier; Chr = chromosome; Pos = position (build 37); OR = odds ratio; CI = confidence interval; PAgediff = P-values for age-difference; PAgejoint = P-values for age-joint test

### Biological follow-up of the additional AMD loci

To refine the causal gene(s) or genetic variant(s) for further prioritizations for functional analyses, the two novel AMD regions were defined as locus regions to be spanned by all variant with r^2^ > 0.5 to the lead variant plus a further 500 kb to each side. For the chromosome 15 locus we identified 1,231 variants and a total of 5 genes, while for the chromosome 18 locus there were 1,313 variants and 6 genes. These were used for our biological and functional follow-up (**Online Methods**): on the variant-level, we derived, (1) the statistically most likely causal variants in each locus using the Bayes factor (**S4 Table**) and (2) their overlap with functional regulatory regions (protein altering, 5’ and 3’ UTR, exonic and promoter regions, **S4 Table**). On the gene-level, we assembled (1) gene expression data from human retina and RPE/choroid cells (**S5 Table)**, and (2) mouse eye phenotypes from the Mouse Genome Informatics data (**S6 Table**). The obtained results were summarized in a gene priority score (GPS) table (**Table 3**). Using equal weights for each column in the table, we observed the highest GPS for the gene encoding the retinaldehyde binding protein 1 (*RLBP1*, GPS = 7) at the chr15-region and the retinal clusterin-like protein *(CLUL1*, GPS = 6) at the chr18-region.

**Table 3:** Gene prioritization scoring for two AMD regions that were undetected by Fritsche et al. We queried 11 genes in the 2 narrow AMD regions (index and proxies, r^2^ ≥ 0.5 and ±500 kb) for biological evidence. Detailed results are shown in the supplement for the expression data (**S5 Table**) and the functional annotation (**S4 Table**) as well as for the mouse data (**S6 Table**).

Locus Name	Chr	Pos_Start	Pos_End	Gene	GPS	Expressed in retina	Expresssed in RPE / Choroid	MGI mouse eye pheno-type	≥1 variant in credible interval set	Protein altering	5' or 3' UTR	other exonic / splice site	Promotor region (+/- 1kbp)	local eQTL (GTEx)	essential gene (based on ExAC)	Mendelian Retinopathy / Maculopathy
*RLBP1*	15	89631380	89745591	*ABHD2*	*7*	+	+	-	+	-	+	+	+	-	+	-
*RLBP1*	15	89753097	89764922	*RLBP1*	***7***	+	+	+	+	-	+	+	+	-	-	0
*RLBP1*	15	89787193	89860362	*FANCI*	*0*	-	-	-	-	-	-	-	-	-	-	-
*RLBP1*	15	89859535	89878026	*POLG*	*3*	+	+	-	-	-	-	-	-	+	-	-
*RLBP1*	15	89869969	89870041	*MIR6766*	*0*	NA	NA	-	-	-	-	-	-	-	NA	-
*CLUL1*	18	319354	500729	*COLEC12*	*3*	+	+	-	-	-	-	-	-	-	+	-
*CLUL1*	18	580368	581524	*CETN1*	*1*	-	-	-	-	-	-	-	-	-	+	-
*CLUL1*	18	596997	650293	*CLUL1*	***6***	+	+	-	+	-	+	+	+	-	-	-
*CLUL1*	18	649619	658340	*C18orf56*	*1*	-	-	-	-	-	-	-	-	-	+	-
*CLUL1*	18	657603	673499	*TYMS*	*1*	-	-	-	-	-	-	-	-	-	+	-
*CLUL1*	18	670323	712662	*ENOSF1*	*2*	+	+	-	-	-	-	-	-	-	-	-

Abbreviations: Chr = chromosome; Pos = position (build 37); GPS = gene priority score; RPE = retinal pigment epithelium; MGI = Mouse Genome Informatics (database); UTR = untranslated region; eQTL = expression quantitative trait locus; GTEx = Genotype-Tissue Expression (database); ExAC = Exome Aggregation Consortium (database); *RLBP1* = Retinaldehyde Binding Protein 1; *CLUL1* = Retinal Clusterin-Like Protein 1; NA = not available;

More specifically, for *RLBP1*, we observed expression in human retinal as well as in human RPE/choroid cells (**Online Methods**, **S5 Table**). Furthermore, *RLBP1* exhibits relevant eye phenotypes in mice (‘retinal degeneration’, ‘decreased retinal photoreceptor cell number’, **S5 Table**). Our Bayesian approach yielded a 63 kb-wide 99% credible set interval covering a total of 51 causal candidate variants at this locus (**S4 Table**). Notably, 36 of these 51 candidate variants are located in the putative regulatory regions of *RLBP1*, including promoter sequences, 5’- or 3’-UTR, exonic or splice site regions (**S4 Table**). Similarly, for the chr18-region *CLUL1* is expressed in retina and RPE/choroid cell lines (**S5 Table**). The 99% credible interval at this locus covers a smaller number of seven likely causal candidate variants (**S4 Table**). Among them, only the lead variant rs9973159 overlaps with a putative regulatory region in the 5’UTR region of the *CLUL1* gene.

### Lack of sex differences in genetic effects of AMD

**Fig 4:**
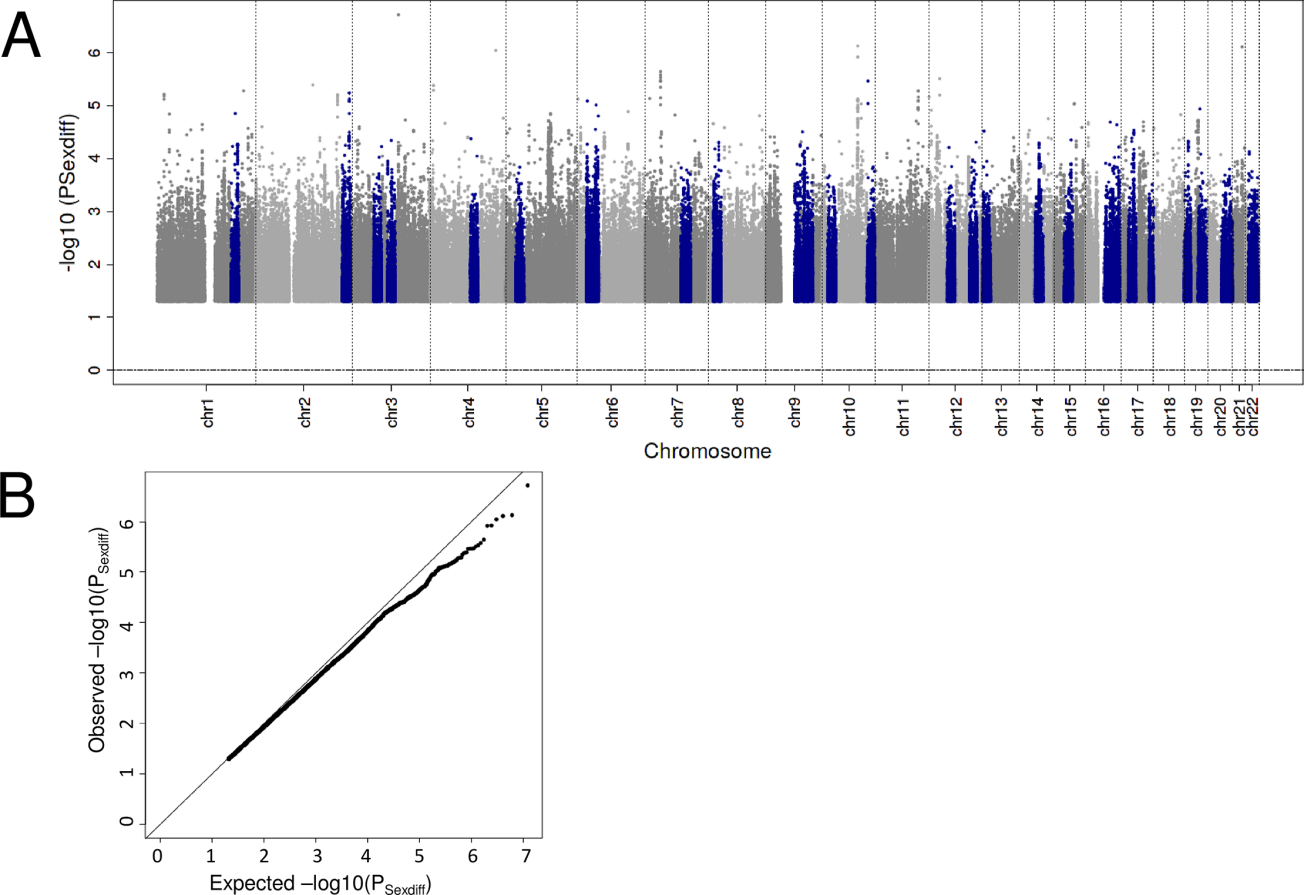
Manhattan and QQ plot of sex-difference P-Values. Shown are the sex-difference P-Values for late AMD by their position on the genome (Manhattan plot) as well as their distribution (QQ plot). The 34 known genetic regions identified by Fritsche et al [13] are colored blue in the Manhattan plot.

It is debated whether women or men have a higher risk of developing late AMD. One might argue that, if the genetic effects explain 47% of the disease variability [13], we can explore whether the 47% of disease etiology bares sex differences. We thus conducted sex-stratified Firth-corrected logistic regression analyses on late AMD in our data set (9,612/10,012 cases/controls among women; 6,532/7,820 cases/controls among men) and tested each variant for sex differences (**Online Methods**). Our genome-wide scan for sex difference failed to reveal variants with a genome-wide significant sex difference (P_Sexdiff_ ≥ 5 x 10^−8^, **Fig 4**). Also, none of the 34 known AMD lead variants yielded significant sex differences in their genetic effects on AMD when judged at a Bonferroni-corrected threshold accounting for the 34 independent tests (P_Sexdiff_ ≥ 0.05/34, **S7 Table**). Noteworthy, we had > 80% power to identify a sex difference to the extent where women exhibit an OR of 1.28 and men lack effect (OR = 1) when judged at genome-wide significance or an OR of 1.22 in women (compared to OR = 1 in men) when judged at 0.05/34. Given the large sample size and thus power of our IAMDGC data set, our null finding suggests that the genetic component of late AMD bares little or no differences between men and women.

## Discussion

Here, we present results of our investigation of age and sex as modulators of genetic effects for late AMD. Our analyses were based on the IAMDGC dataset [13], the currently largest known study on late AMD genetics. We have made three important observations. Firstly, we provide evidence for age to modulate genetic effects. The *CFH* and the *ARMS2/HTRA1* locus, which are the two regions with the largest association signals for late AMD, revealed a larger genetic relative risk in the younger individuals. We found no evidence of qualitative interaction, i.e. no variant effect was restricted to one of the age-groups or was protective in one age-group and adverse in the other. Secondly, by accounting for potentially differential genetic effects between age groups, we identified two AMD loci that were undetected in a previous main effect screen using the identical dataset [13]. These two additional AMD loci point to a novel AMD GWAS region on chromosome 15 and one region on chromosome 18 that was recently identified as AMD risk locus in a joint analysis of nuclear and mitochondrial variants [20]. Thirdly, we found no differences in the genetic effects for late AMD between men and women despite considerable power in our study design.

The finding of two additional AMD loci is interesting in two-ways: functionally and methodologically. Functionally, in each of the two loci we identified plausible genes conferring susceptibility to late AMD, the *RLBP1* and the *CLUL1* gene. There were 11 gene candidates in the two chromosomal regions at chromosome 15 and 18. Following a systematic approach summarizing biological and functional evidence as applied previously [13], we yielded the highest evidence for *RLBP1* and *CLUL1* in the two loci, respectively. Previous literature strongly supports a functional connection of each of these two genes to the visual system and their role in retinal disease: *RLBP1* is a functional component of the “visual cycle” and mutations in the *RLBP1* gene have been associated with autosomal recessive rod-cone dystrophies [21], such as autosomal recessive retinitis pigmentosa [22], Bothnia dystrophy [23,24], Newfoundland rod-cone dystrophy [25], retinitis punctata albescens [26,27], and fundus albipunctatus [26] (**S8 Table**). *CLUL1* is a cone photoreceptor-specific gene under cone-rod homeobox (CRX) regulation; its protein, known as retinal clusterin-like protein 1, shows light-dependent translocation, i.e. in light-adapted retina, *CLUL1* has been found in the outer segment of cone photoreceptors while in dark-adapted retina, protein expression was demonstrated in the contact region between cone pedicles and second-order neurons [28–30]. This indicates that *CLUL1* is likely to be critical for normal cone function [30]. Furthermore, *CLUL1* transcripts are developmentally regulated in parallel with retinal differentiation, suggesting a functional role during photoreceptor differentiation [30]. Based on its developmental regulation, distinct localization, and possible involvement in a wide range of cellular retinal processes, *CLUL1* represents a potential candidate for retinal diseases, particularly those that affect cones. Moreover, clusterin has been found to be a common protein identified in drusen preparations from explanted retinae of AMD donor eyes [30]. On this basis, *CLUL1* was previously considered a candidate gene for AMD and a mutation screen of the coding region of the *CLUL1* gene in unrelated patients with AMD was reported [30]. Importantly, in the *CLUL1* locus, the 99% credible set of associated variants is comprised of a single variant (rs9973159) in the 5’UTR of the gene. This is important for future functional studies, since this single variant is statistically the likely true causal variant and potentially influences gene expression either by modulating promotor activity or by influencing transcript stability. Methodologically, the identification of novel loci with small gene-age interaction illustrates the ability of the joint test to leverage such interactions in a genome-wide search. The two loci were missed in previous main effect scans, including our own previous analysis utilizing the identical data set but without accounting for potential age differences [13].

From our refined analysis, what is the lesson learned about the etiology of late AMD? Age is the strongest risk factor for late AMD together with the joint genetic profile. The disease variability explained by genetic variants was reported to be as high as 47% [13]. One question that arises is whether the identified factors age and genetics act independently or whether there is a joint component with interacting effects. Such a shared etiology includes genetic effects that appear more pronounced in the younger or in the older. The prior seem to point towards genetic effects that are attenuated by additional factors related to ageing, while the latter effects could be directly related to genetic factors that modulate aging processes. Such interaction effects would also include genetic effects with opposite directions, i.e. effects that are protective in younger and adverse in older individuals or vice versa, which we did not observe in our data despite considerable power. Our results indicate genetic loci with more pronounced effects in the younger than in the older, which is specifically true for variants in the *CFH*, *ARMS/HTRA*, *RLBP1*, and *CLUL1* loci. This is in line with the observation that the cumulative genetic risk for late AMD calculated for 13 genetic variants was higher in younger than in older individuals [14]. For the *RLBP1* locus, the stronger effect on AMD in the younger is linked to the observed modification of the effect of this variant to AMD disease development (or a highly correlated variant, rs11459118, r^2^ = 0.85) given the genotypes of a mitochondrial variant [20]. One might speculate that a genetically modified mitochondrial function triggers the effect of the nuclear variant in the younger, but may not cause a similar damage in the older due to a well-known decline of mitochondrial function at higher age [31].

Genes located in GWAS loci are twice as effective in drug development pipelines as random genes [32]. The question that arises is: What can we learn from gene-age interaction, or a lack thereof, for a gene’s potential to be a successful drug target. Genes with quantitative gene-age interaction, i.e. differentially pronounced effects by age groups that point to the same effect direction, can be assumed to be potential drug targets. Therefore, a genomic screen accounting for age as potential modulator, as conducted in the present study, can effectively complement the drug target list. Genes with zero effect in either the younger or the older group might be puzzling and an investigation of the reasons for such effects might help understand underlying mechanisms. Genes with truly qualitative gene-age interaction to the extent that there is a protective effect in one age-group and an adverse effect on another age-group pose the question of the uncertainty in the age cut-off for drug indication. This can be cumbersome and expensive in drug development.

Also in light of a potentially modifying role of sex on late AMD risk, our results contribute to the ongoing debate [3]. Our systematic scan failed to reveal a significant sex difference in any variant genome-wide, despite sufficient power of our analysis to detect a difference where the relative risk is as high as 1.28 or higher in women and 1.0 (null effect) in men (or vice versa). Also for the lead variants in the 34 loci, we found no sex difference, and here our power was sufficient to detect a difference for a relative risk of 1.22 or higher in women and 1.0 in men (or vice versa). Since the previously published variants alone explain 47% of the late AMD cases in our data set [13], we may conclude that, at least in this ~47% of disease etiology, there is no difference between men and women in the probability of developing late AMD.

Ideally, the interaction of genetic effects and age should be evaluated in longitudinal data. However, the effective sample size, which is determined by the number of late AMD cases occurring during the follow-up, of such a longitudinal study available so far is < 500 [33]. On the other hand, the question can also be addressed in population-based cross-sectional data when assuming little cohort effects, i.e. differences in the individuals that were born many decades ago compared to individuals born more recently [34]. There are larger sample sizes available in such cross-sectional studies, but a cross-sectional study data with > 10.000 late AMD cases with an estimated prevalence of 1% among general adults would need to be as large as 1 million, which is not available for cross-sectional study data with genome-wide information at the current time. The case-control setting of our data with 17,000 late AMD cases overcomes this problem constituting the largest data on late AMD genetics to date. However, this does come at a price. Absolute risk from age, genetics, and the share between age and genetics cannot be estimated. Another potential drawback is the uncertainty in the age that is used in this analysis. Ideally, this would be age-of-onset for late AMD and the age distribution of controls should be fully matching the age distribution of patients. For our late AMD patients, the participants’ “age” was determined as the age at first exam when late AMD has been diagnosed. For control subjects, it is the age at last exam, when the individual was found to be AMD-free. Thus, the “age” estimate for our late AMD patients can be considered to be left-censored (i.e. the age-of-onset is at least as large as observed, but can be smaller). Our controls’ age distribution is similar to the cases, including individuals as old as 101 years, but also includes some younger individuals with < 50 years. We conducted a sensitivity analysis excluding control subjects below the age of 50 [13], which had no impact on the genetic effect sizes of the 34 late AMD variants. From this, we conclude that this issue is rather minor and should not affect our conclusions. Beside the large sample size of our investigation, the strength of our data was the centrally genotyped data on a single chip for all included subjects. A further strength is our systematic approach to evaluate gene x age and gene x sex interaction for late AMD genome-wide rather than applying a candidate gene approach.

Our investigation using the largest dataset on late AMD genetics to date, revealed evidence for genetic effects on late AMD that are stronger in the younger compared to the older. We found no evidence for qualitative gene x age interaction or any role of sex in the effects of late AMD genetics. Importantly, we detected two additional genome-wide significant loci for late AMD compared to our previous analysis, which include a compelling gene in each of these, *RLBP1* and *CLUL1*, as relevant for late AMD. These two genes offer plausible and possibly actionable targets for further investigation.

## Methods

### Ethics statement

The Institutional Review Board (IRB) of the University of Utah was the umbrella IRB for all other studies contributing data to the International Age-related Macular Degeneration Genomics Consortium (IAMDGC), except for the Beaver Dam Eye Study (BDES). The University of Utah approved and certified each individual study ethic committee's conduct for the data used in this study. Data provided by BDES was approved by the IRB of the University of Wisconsin.

### Study data acquisition

We based our analyses on individual participant data from 26 studies of the International AMD Genomic Consortium (IAMDGC) [13]. This data comprised genotype information from 16,144 AMD cases and 17,832 controls after quality control. The genotype data was derived and quality controlled centrally using a customized Illumina HumanCoreExome array that contains genome-wide content, exome content (up to 163,714 mostly rare, protein-altering variants) as well as fine-mapping variants for 22 previously known AMD loci [35]. Unmeasured genotypes were imputed centrally by IAMDGC analysts to the 1000 Genomes phase 1 version 3 reference panel yielding >12 million variants for the association analyses. Details on the aggregation of data, genotyping and imputation as well as quality control are described in detail elsewhere [13].

### Stratified association analyses and quality control

In order to analyze interaction of a genetic factor with a dichotomous exposure variable, there are two statistical concepts, modeling with an interaction term or stratifying for the dichotomous exposure variable (i.e. high/low age, female/male sex) and comparing the genetic effects across strata [36]. Both approaches enable the testing of a genetic effect for difference between the two groups and an accounting for a potential interaction in the search for a genetic effect [37]. The stratified approach has some advantage when there are other covariates in the model as it does not make any assumptions about these covariates’ association with any of the other covariates, while the interaction term modeling either makes assumptions or it includes interaction terms with each of the other covariates, including three- or four-way interactions, which make the models basically equivalent to a stratified model, but less intuitive to interpret [36]. We thus conducted age-group-stratified as well as sex-stratified genome-wide association analyses based on the IAMDGC data. For the age-stratified analyses, we separated the IAMDGC data into two age-groups that were defined by the median of age among cases = 77.8 years of age) and by the median of age among controls = 71.0 years of age). Our age-stratification yielded 7,959 and 9,072 younger cases (≤ 77.8y) and controls (≤ 71.0y), respectively, as well as 7,934 and 8,653 older cases (> 77.8y) and controls (> 71.0y), respectively. Stratification of the IAMDGC data by sex yielded 6,532 and 7,820 male cases and controls, respectively, as well as 9,612 and 10,012 female cases and controls, respectively. For each subgroup, i.e., for younger and older, men and women, separately, we conducted a genome-wide association scan. We applied Firth-bias corrected logistic regression analyses to each variant and included the first two principal components as well as whole genome amplification status as covariates in the regression models as implemented previously [13]. Variants with minor allele count less than 20 were excluded from the stratified association analyses and genomic control correction was applied to correct for potential population stratification or relatedness across individuals. We excluded variants harboring any of the known 34 AMD loci (+/- 10Mb around previously published AMD loci) for the calculation of the genomic control inflation factor. We observed low inflation factors for the genome-wide association results in the younger (λ_GC,≤50y_ = 1.08), the older (λ_GC,>50y_ = 1.03), the men (λ_GC,Men_ = 1.04) and the women (λ_GC,Women_ = 1.06).

### Testing for differences in genetic effects on late AMD

We utilized age- and sex-specific association scan results to identify differences in genetic effects on AMD between younger and older individuals as well as between men and women. For each variant, we implemented a Z-Test to compare age-stratified effects for difference between the younger and the older participants:

$$Z_{Agediff}=\frac{{\hat{\beta }}_{Y}-{\hat{\beta }}_{O}}{\sqrt{se_{y}^{2}+se_{o}^{2}-2r_{Age}se_{Y}se_{O}}}.$$ (1)

Here, ${\hat{\beta }}_{Y}$ and ${\hat{\beta }}_{O}$ reflect the age-specific effect sizes (log odds-ratios) with standard errors *se*_*Y*_ and *se*_*O*_, estimated from the age-stratified regression models, and *r*_*Age*_ reflects the Spearman rank correlation coefficient between the effect sizes of the younger and the older individuals (*r*_*Age*_ = 0.03, estimated from the IAMDGC data). Analogously, we applied a Z Test to compare sex-stratified effects for difference between male and female sex (*r*_*Sex*_ = 0.03, estimated from the IAMDGC data):

$$Z_{Sexdiff}=\frac{{\hat{\beta }}_{M}-{\hat{\beta }}_{F}}{\sqrt{se_{M}^{2}+se_{F}^{2}-2r_{Sex}se_{M}se_{F}}}.$$ (2)

In order to conduct a genome-wide search for genetic effects that differ by age or those that differ by sex (i.e. search for GxAGE and GxSEX), we applied the difference tests to all variants genome-wide and selected variants with significantly different effect sizes using a genome-wide significance level (*P*_*Agediff*_ < 5 x 10^−8^ to declare significant age-difference, *P*_*Sexdiff*_ < 5 x 10^−8^ to declare significant sex-difference). This approach has been shown to increase the power to detect genetic effects with opposite effects in the two groups of interest and effects [37]. Besides this hypothesis-free approach to search for differences genome-wide, we also conducted a focused follow-up of the 34 known AMD lead variants (i.e. testing known variants for GxAGE or GxSEX). We thus tested the 34 lead variants’ effects on late AMD for age-differences and for sex-difference using a Bonferroni-corrected significance threshold (P_Agediff_ < 0.05/34 to declare significant age-difference, or P_Sexdiff_ < 0.05/34 to declare significant sex-difference).

### Identification of novel AMD regions by testing for joint stratified genetic effects

In order to explore whether we could detect novel late AMD loci by accounting for potential interaction of the genetic effect with age or sex (i.e. search for G accounting for GxAGE or GxSEX), we jointly tested the age-stratified effects as well as the sex-stratified effects for association using a 2 degrees-of-freedom (2df) chi-squared test [19]. A genome-wide screen using this test is known to increase power to identify associated regions when there are some variants with differences between the two groups. We thus applied the following 2df joint tests to the age-stratified and to the sex-stratified effects of each variant:

$$C_{Agejoint}={\left(\frac{{\hat{\beta }}_{Y}}{se_{Y}}\right)}^{2}+{\left(\frac{{\hat{\beta }}_{O}}{se_{O}}\right)}^{2},$$ (3)

$$C_{Sexjoint}={\left(\frac{{\hat{\beta }}_{M}}{se_{M}}\right)}^{2}+{\left(\frac{{\hat{\beta }}_{F}}{se_{F}}\right)}^{2}.$$ (4)

We conducted a hypothesis-free approach and screened all variants for potential joint 2df effects using a genome-wide significance level (P_Agejoint_ < 5 x 10^−8^ to declare significant joint 2df age-stratified effects, and P_Sexjoint_ < 5 x 10^−8^ to declare significant joint 2df sex-stratified effects).

### Clumping of genome-wide significant variants into independent regions and conditional analyses to define independent signals

We clumped each set of genome-wide significant variants (either showing age-difference, sex-difference, joint age-stratified or joint sex-stratified effects) into independent regions using a liberal physical distance threshold of +/-10M base positions. For each region, the variant with the smallest P-Value (*P*_*Agediff*_, *P*_*Sexdiff*_, *P*_*Agejoint*_, or *P*_*Sexjoint*_, respectively) was defined to be the lead variant. To identify additional independent signals within regions with significant differences or within novel AMD regions with significant joint effects, the stratified association analyses were repeated for all variants of the respective region while conditioning on the lead variant. We then tested the conditioned stratified effects for differences or for joint effects and selected any variant showing conditional genome-wide significance (P_Agediff,Cond_ < 5 x 10^−8^, P_Sexdiff,Cond_ < 5 x 10^−8^, P_Agejoint,Cond_ < 5 x 10^−8^, or P_Sexjoint,Cond_ < 5 x 10^−8^, respectively). We repeated the procedure until no additional signal was identified. At each novel identified region, a locus definition was applied according to Fritsche et al 2016 [13]. Locus regions were defined by extracting all variants that are correlated with the lead variant (r^2^>0.5) and by adding a further 500 kb to both sides. Variants and genes overlapping the so-defined locus regions were considered as candidate variants and candidate genes and were used for biological follow-up analyses. Regional association plots of the identified novel regions were created using Locuszoom (**http://locuszoom.sph.umich.edu/**) [38].

### Functional follow-up of newly identified loci

In order to prioritize genes in the newly identified late AMD loci, we investigated gene expression, known mouse phenotype related to AMD, derived the most likely causal variants in the loci and evaluated their role for regulatory function.

1. Expression of candidate genes in retina and RPE/choroid was assessed using Next-Generation transcriptome sequencing as described previously [13]. Genes with fragments per kilobase exonic sequence per million reads mapped (FPKM) value greater than one were deemed to be expressed in the respective tissue [39].
2. The Mouse Genome Informatics (MGI) database (www.informatics.jax.org/) was queried for the candidate genes and results were evaluated for relevant eye and high-level phenotypes in established genetic mouse models.
3. A Bayesian approach to prioritize causal variants at novel locus regions was applied. Herewith, the Bayes Factor based posterior probability of each variant was computed using association z scores according to Kichaev et al [40]. The method assumes that there is precisely one causal signal and cannot be applied to regions covering multiple independent signals. We derived the 99% credible intervals for each of the novel locus regions as applied previously [41].
4. To further explore any regulatory function of variants in the loci, we used the variant effect predictor from Ensembl [42] to assess the functional impact of the variants in the 99% credible set on canonical transcripts. In addition, we used the Genotype-Tissue Expression (GTEx) database [43] to assess if any of the credible variants is a local eQTL for one of the candidate genes in any tissue included in GTEx. Although no retinal tissues are currently included in the GTEx database, recent findings indicate that the majority of local eQTL are shared across tissues [44]. Next, we assessed whether a gene can be considered to be essential for human survival, i.e. does not tolerate loss of function mutations using the Exome Aggregation Consortium (ExAC) database [45]. Finally, we investigated if any of the candidate genes is frequently mutated in inherited (Mendelian) retinopathies or maculopathies using the RetNet database [46].

## Supporting information

S1 Table Age-specific AMD association results for the 34 lead variants from Fritsche et al. 2016. Variants with significant difference between younger and older individuals (P_Agediff_ < 0.05/34) are marked in bold and presented in **Table 1**.
(XLSX)

S2 Table Sensitivity age-stratified analysis for the three variants with significant age-difference. The table shows results from a sensitivity age-stratified analysis based on a stratification of cases into truly younger cases (< = 65.0y) and truly older cases (> = 85.0y). Controls were stratified as before by median of age within controls = 71.0y). Consistent with the primary age-stratified analyses, genetic effects among younger individuals are larger than genetic effecst among older individuals. One variant misses significance on the age-difference P Value due to the lower number of cases and thus lower power of the sensitivity analysis.
(XLSX)

S3 Table Age-specific AMD association results for 29 lead variants with genome-wide significant age-joint effects (P_Agejoint_<5 x 10–8). The two variants that were not detected by Fritsche et al 2016 are also shown in **Table 2.**
(XLSX)

S4 Table Credible set variants (CI>99%) and annotation of putative regulatory location for the two additional regions with genome-wide significant age-joint effects. (XLSX)

S5 Table Expression results from Weber lab. The Fragments per kilobase of exonic sequence per million reads mapped (FPKM) of candidate genes in different retinal tissues are shown. We considered genes with an FPKM value greater than 1 to be expressed in a tissue (either RPE/choroid or Retina).
(XLSX)

S6 Table Mouse phenotype lookups (MGI data) for the candidate genes at the two additional AMD regions. (XLSX)

S7 Table Sex-specific association results for late AMD and for the 34 lead variants from Fritsche et al 2016. (XLSX)

S8 Table Details on RLBP1-associated autosomal recessive rod-cone disorders. Listed are known mutations (extracted from https://www.ncbi.nlm.nih.gov/clinvar?term=180090[MIM], November 2017), epidemiological information as well as ophthalmological key features. Please note that the clinical distinction between some of these diseases may be subtle and dependent on the age at diagnosis.
(XLSX)

## References

[1] Augood CA, Vingerling JR, de Jong PT, Chakravarthy U, Seland J, et al (2006) Prevalence of age-related maculopathy in older Europeans: the European Eye Study (EUREYE). Arch Ophthalmol 124: 529–535. doi: 10.1001/archopht.124.4.529 16606879 10.1001/archopht.124.4.529

[2] Lim LS, Mitchell P, Seddon JM, Holz FG, Wong TY (2012) Age-related macular degeneration. Lancet 379: 1728–1738. doi: 10.1016/S0140-6736(12)60282-7 22559899 10.1016/S0140-6736(12)60282-7

[3] Yonekawa Y, Miller JW, Kim IK (2015) Age-Related Macular Degeneration: Advances in Management and Diagnosis. J Clin Med 4: 343–359. doi: 10.3390/jcm4020343 26239130 10.3390/jcm4020343 PMC4470128

[4] Rosenfeld PJ (2011) Bevacizumab versus ranibizumab for AMD. N Engl J Med 364: 1966–1967. doi: 10.1056/NEJMe1103334 21526924 10.1056/NEJMe1103334

[5] (2000) Risk factors associated with age-related macular degeneration. A case-control study in the age-related eye disease study: Age-Related Eye Disease Study Report Number 3. Ophthalmology 107: 2224–2232. 11097601 10.1016/s0161-6420(00)00409-7 PMC1470467

[6] Smith W, Assink J, Klein R, Mitchell P, Klaver CC, et al (2001) Risk factors for age-related macular degeneration: Pooled findings from three continents. Ophthalmology 108: 697–704. 11297486 10.1016/s0161-6420(00)00580-7

[7] Klein R, Klein BE, Jensen SC, Meuer SM (1997) The five-year incidence and progression of age-related maculopathy: the Beaver Dam Eye Study. Ophthalmology 104: 7–21. 9022098 10.1016/s0161-6420(97)30368-6

[8] Mitchell P, Smith W, Attebo K, Wang JJ (1995) Prevalence of age-related maculopathy in Australia. The Blue Mountains Eye Study. Ophthalmology 102: 1450–1460. 9097791 10.1016/s0161-6420(95)30846-9

[9] Friedman DS, O'Colmain BJ, Munoz B, Tomany SC, McCarty C, et al (2004) Prevalence of age-related macular degeneration in the United States. Arch Ophthalmol 122: 564–572. doi: 10.1001/archopht.122.4.564 15078675 10.1001/archopht.122.4.564

[10] Klein R, Klein BE, Linton KL (1992) Prevalence of age-related maculopathy. The Beaver Dam Eye Study. Ophthalmology 99: 933–943. 1630784 10.1016/s0161-6420(92)31871-8

[11] Vingerling JR, Dielemans I, Hofman A, Grobbee DE, Hijmering M, et al (1995) The prevalence of age-related maculopathy in the Rotterdam Study. Ophthalmology 102: 205–210. 7862408 10.1016/s0161-6420(95)31034-2

[12] Fraser-Bell S, Donofrio J, Wu J, Klein R, Azen SP, et al (2005) Sociodemographic factors and age-related macular degeneration in Latinos: the Los Angeles Latino Eye Study. Am J Ophthalmol 139: 30–38. doi: 10.1016/j.ajo.2004.08.029 15652825 10.1016/j.ajo.2004.08.029

[13] Fritsche LG, Igl W, Bailey JN, Grassmann F, Sengupta S, et al (2016) A large genome-wide association study of age-related macular degeneration highlights contributions of rare and common variants. Nat Genet 48: 134–143. doi: 10.1038/ng.3448 26691988 10.1038/ng.3448 PMC4745342

[14] Grassmann F, Fritsche LG, Keilhauer CN, Heid IM, Weber BH (2012) Modelling the genetic risk in age-related macular degeneration. PLoS One 7: e37979 doi: 10.1371/journal.pone.0037979 22666427 10.1371/journal.pone.0037979 PMC3364197

[15] Naj AC, Scott WK, Courtenay MD, Cade WH, Schwartz SG, et al (2013) Genetic factors in nonsmokers with age-related macular degeneration revealed through genome-wide gene-environment interaction analysis. Ann Hum Genet 77: 215–231. doi: 10.1111/ahg.12011 23577725 10.1111/ahg.12011 PMC3625984

[16] Baird PN, Robman LD, Richardson AJ, Dimitrov PN, Tikellis G, et al (2008) Gene-environment interaction in progression of AMD: the CFH gene, smoking and exposure to chronic infection. Hum Mol Genet 17: 1299–1305. doi: 10.1093/hmg/ddn018 18203751 10.1093/hmg/ddn018

[17] Seddon JM, George S, Rosner B, Klein ML (2006) CFH gene variant, Y402H, and smoking, body mass index, environmental associations with advanced age-related macular degeneration. Hum Hered 61: 157–165. doi: 10.1159/000094141 16816528 10.1159/000094141

[18] Kraft P, Yen YC, Stram DO, Morrison J, Gauderman WJ (2007) Exploiting gene-environment interaction to detect genetic associations. Hum Hered 63: 111–119. doi: 10.1159/000099183 17283440 10.1159/000099183

[19] Aschard H, Hancock DB, London SJ, Kraft P (2010) Genome-wide meta-analysis of joint tests for genetic and gene-environment interaction effects. Hum Hered 70: 292–300. doi: 10.1159/000323318 21293137 10.1159/000323318 PMC3085519

[20] Persad PJ, Heid IM, Weeks DE, Baird PN, de Jong EK, et al (2017) Joint Analysis of Nuclear and Mitochondrial Variants in Age-Related Macular Degeneration Identifies Novel Loci TRPM1 and ABHD2/RLBP1. Invest Ophthalmol Vis Sci 58: 4027–4038. doi: 10.1167/iovs.17-21734 28813576 10.1167/iovs.17-21734 PMC5559178

[21] Hipp S, Zobor G, Glockle N, Mohr J, Kohl S, et al (2015) Phenotype variations of retinal dystrophies caused by mutations in the RLBP1 gene. Acta Ophthalmol 93: e281–286. doi: 10.1111/aos.12573 25429852 10.1111/aos.12573

[22] Maw MA, Kennedy B, Knight A, Bridges R, Roth KE, et al (1997) Mutation of the gene encoding cellular retinaldehyde-binding protein in autosomal recessive retinitis pigmentosa. Nat Genet 17: 198–200. doi: 10.1038/ng1097-198 9326942 10.1038/ng1097-198

[23] Burstedt MS, Sandgren O, Holmgren G, Forsman-Semb K (1999) Bothnia dystrophy caused by mutations in the cellular retinaldehyde-binding protein gene (RLBP1) on chromosome 15q26. Invest Ophthalmol Vis Sci 40: 995–1000. 10102298

[24] He X, Lobsiger J, Stocker A (2009) Bothnia dystrophy is caused by domino-like rearrangements in cellular retinaldehyde-binding protein mutant R234W. Proc Natl Acad Sci U S A 106: 18545–18550. doi: 10.1073/pnas.0907454106 19846785 10.1073/pnas.0907454106 PMC2774026

[25] Eichers ER, Green JS, Stockton DW, Jackman CS, Whelan J, et al (2002) Newfoundland rod-cone dystrophy, an early-onset retinal dystrophy, is caused by splice-junction mutations in RLBP1. Am J Hum Genet 70: 955–964. doi: 10.1086/339688 11868161 10.1086/339688 PMC379124

[26] Katsanis N, Shroyer NF, Lewis RA, Cavender JC, Al-Rajhi AA, et al (2001) Fundus albipunctatus and retinitis punctata albescens in a pedigree with an R150Q mutation in RLBP1. Clin Genet 59: 424–429. 11453974 10.1034/j.1399-0004.2001.590607.x

[27] Morimura H, Berson EL, Dryja TP (1999) Recessive mutations in the RLBP1 gene encoding cellular retinaldehyde-binding protein in a form of retinitis punctata albescens. Invest Ophthalmol Vis Sci 40: 1000–1004. 10102299

[28] Zhang Q, Beltran WA, Mao Z, Li K, Johnson JL, et al (2003) Comparative analysis and expression of CLUL1, a cone photoreceptor-specific gene. Invest Ophthalmol Vis Sci 44: 4542–4549. 14507903 10.1167/iovs.02-1202

[29] Zhang Q, Ray K, Acland GM, Czarnecki JM, Aguirre GD (2000) Molecular cloning, characterization and expression of a novel retinal clusterin-like protein cDNA. Gene 243: 151–160. 10675623 10.1016/s0378-1119(99)00542-9

[30] Sturgill GM, Pauer GJ, Bala E, Simpson E, Yaniglos SS, et al (2006) Mutation screen of the cone-specific gene, CLUL1, in 376 patients with age-related macular degeneration. Ophthalmic Genet 27: 151–155. doi: 10.1080/13816810600976871 17148042 10.1080/13816810600976871 PMC3021946

[31] Sun N, Youle RJ, Finkel T (2016) The Mitochondrial Basis of Aging. Mol Cell 61: 654–666. doi: 10.1016/j.molcel.2016.01.028 26942670 10.1016/j.molcel.2016.01.028 PMC4779179

[32] Nelson MR, Tipney H, Painter JL, Shen J, Nicoletti P, et al (2015) The support of human genetic evidence for approved drug indications. Nat Genet 47: 856–860. doi: 10.1038/ng.3314 26121088 10.1038/ng.3314

[33] Buitendijk GH, Rochtchina E, Myers C, van Duijn CM, Lee KE, et al (2013) Prediction of age-related macular degeneration in the general population: the Three Continent AMD Consortium. Ophthalmology 120: 2644–2655. doi: 10.1016/j.ophtha.2013.07.053 24120328 10.1016/j.ophtha.2013.07.053 PMC3986722

[34] (2017) Generational Differences in the 5-Year Incidence of Age-Related Macular Degeneration. JAMA Ophthalmol 135: 1417–1423. doi: 10.1001/jamaophthalmol.2017.5001 29145549 10.1001/jamaophthalmol.2017.5001 PMC5902189

[35] Fritsche LG, Chen W, Schu M, Yaspan BL, Yu Y, et al (2013) Seven new loci associated with age-related macular degeneration. Nat Genet 45: 433–439, 439e431-432. doi: 10.1038/ng.2578 23455636 10.1038/ng.2578 PMC3739472

[36] Sung YJ, Winkler TW, Manning AK, Aschard H, Gudnason V, et al (2016) An Empirical Comparison of Joint and Stratified Frameworks for Studying G x E Interactions: Systolic Blood Pressure and Smoking in the CHARGE Gene-Lifestyle Interactions Working Group. Genet Epidemiol 40: 404–415. doi: 10.1002/gepi.21978 27230302 10.1002/gepi.21978 PMC4911246

[37] Winkler TW, Justice AE, Cupples LA, Kronenberg F, Kutalik Z, et al (2017) Approaches to detect genetic effects that differ between two strata in genome-wide meta-analyses: Recommendations based on a systematic evaluation. PLoS One 12: e0181038 doi: 10.1371/journal.pone.0181038 28749953 10.1371/journal.pone.0181038 PMC5531538

[38] Pruim RJ, Welch RP, Sanna S, Teslovich TM, Chines PS, et al (2010) LocusZoom: regional visualization of genome-wide association scan results. Bioinformatics 26: 2336–2337. doi: 10.1093/bioinformatics/btq419 20634204 10.1093/bioinformatics/btq419 PMC2935401

[39] Brandl C, Grassmann F, Riolfi J, Weber BH (2015) Tapping Stem Cells to Target AMD: Challenges and Prospects. J Clin Med 4: 282–303. doi: 10.3390/jcm4020282 26239128 10.3390/jcm4020282 PMC4470125

[40] Kichaev G, Yang WY, Lindstrom S, Hormozdiari F, Eskin E, et al (2014) Integrating functional data to prioritize causal variants in statistical fine-mapping studies. PLoS Genet 10: e1004722 doi: 10.1371/journal.pgen.1004722 25357204 10.1371/journal.pgen.1004722 PMC4214605

[41] Grassmann F, Heid IM, Weber BH, International AMDGC (2017) Recombinant Haplotypes Narrow the ARMS2/HTRA1 Association Signal for Age-Related Macular Degeneration. Genetics 205: 919–924. doi: 10.1534/genetics.116.195966 27879347 10.1534/genetics.116.195966 PMC5289859

[42] Yates A, Akanni W, Amode MR, Barrell D, Billis K, et al (2016) Ensembl 2016. Nucleic Acids Res 44: D710–716. doi: 10.1093/nar/gkv1157 26687719 10.1093/nar/gkv1157 PMC4702834

[43] Consortium GT (2015) Human genomics. The Genotype-Tissue Expression (GTEx) pilot analysis: multitissue gene regulation in humans. Science 348: 648–660. doi: 10.1126/science.1262110 25954001 10.1126/science.1262110 PMC4547484

[44] Aguet F, Brown AA, Castel S, Davis JR, Mohammadi P, et al (2016) Local genetic effects on gene expression across 44 human tissues. Cold Spring Harbor Labs Journals bioRxiv.

[45] Lek M, Karczewski KJ, Minikel EV, Samocha KE, Banks E, et al (2016) Analysis of protein-coding genetic variation in 60,706 humans. Nature 536: 285–291. doi: 10.1038/nature19057 27535533 10.1038/nature19057 PMC5018207

[46] Daiger S, Rossiter B, Greenberg J, Christoffels A, Hide W (1998) RetNet—Retinal Information Network 1998. Data services and software for identifying genes and mutations causing retinal degeneration. Invest OphthalmolVis Sci 39: 295.

